# Supplementary material for: The HIV Restriction Factor Profile in the Brain Is Associated with the Clinical Status and Viral Quantities
Source: Viruses. 2023 Jan 23;15(2):316. doi: 10.3390/v15020316 (PMC9962287; doi:10.3390/v15020316)
Supplement: Supplementary file 1 [file viruses-15-00316-s001.zip › viruses-2145333-supplementary.pdf]

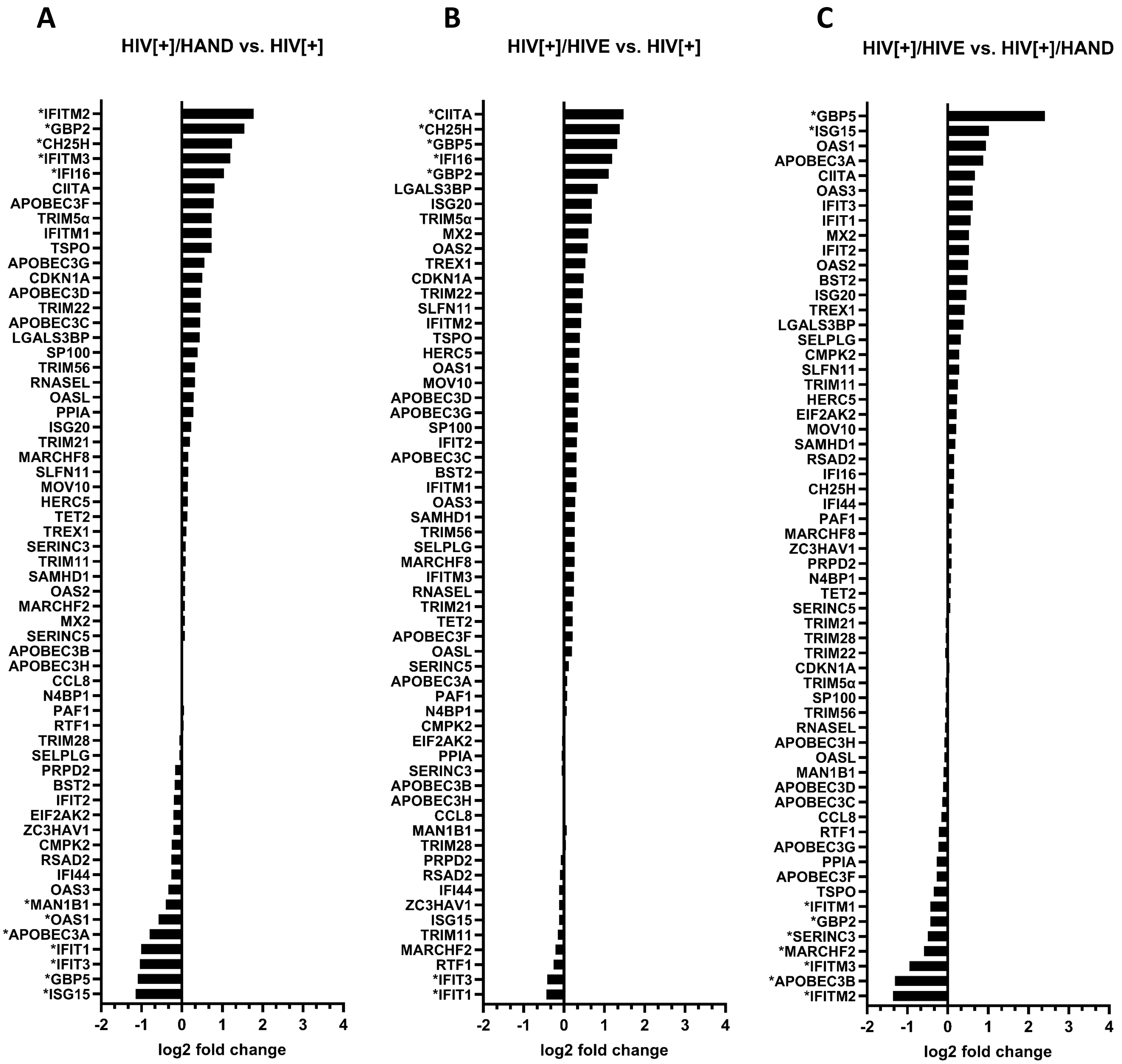

Figure S1. ***Relative host restriction factors' mRNA expression levels in RNA-seq database.*** Sixty known host restriction factor genes with antiretroviral capabilities were screened in the RNA-seq dataset. Differentially expressed genes are reported in different group comparisons. (A) HIV[+]/HAND compared to HIV[+], (B) HIV[+]/HIVE compared to HIV[+], and (C) HIV[+]/HIVE compared to HIV[+]/HAND. Fold changes range from -2, -1, 0, 1, 2, 3, 4 log2 fold change (or 0.25, 0.5, 1, 2, 4, 8, 16-fold changes). Genes with 0 to -2 log2 fold change are considered downregulated and 0 to 4 log2 fold change are considered upregulated). Asterisks indicate the gene was selected for validation by q RT-PCR.

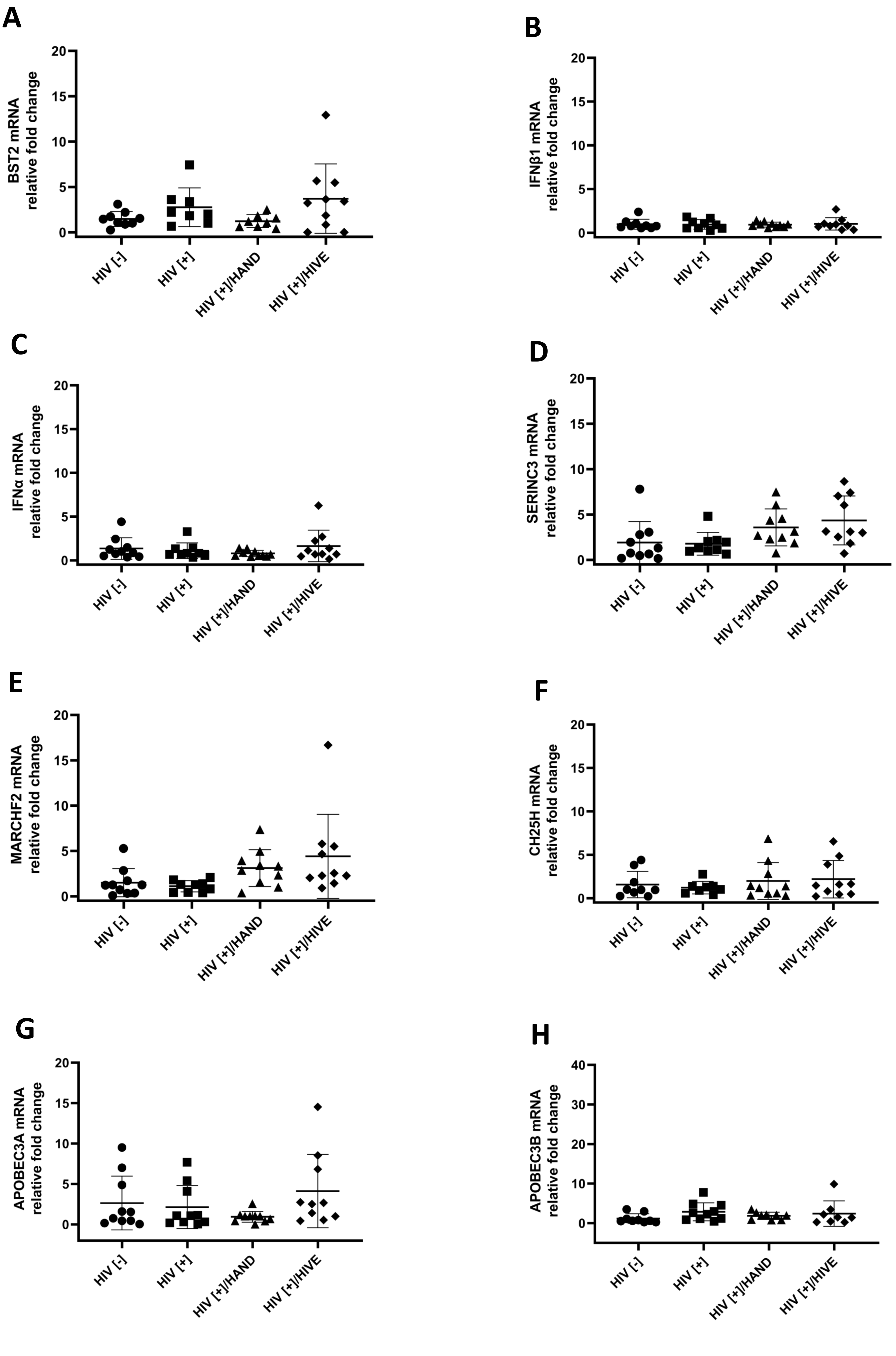

Figure S2. ***Relative restriction factors' mRNA expression levels in human brains.*** qRT-PCR analyses identified upregulated transcripts during HIV infection and/or HAND by RNA-seq; *BST2* showed modest upregulation in HIV[+] and HIV[+] HAND groups (A). *IFNA* and *IFNB1* levels were similar across the clinical groups (B and C). Q RT-PCR analysis of downregulated genes (identified by RNA seq) *SERINC3* (D) and *MARCHF2* (E) did not support RNA-seq's suggested downregulation in HIVE group compared to HAND. The same trend was observed for *CH25H* (F), *APOBEC3A* (G), and *APOBEC3B* (H) transcript levels. The horizontal lines represent mean values, and the error bars represent standard deviation.

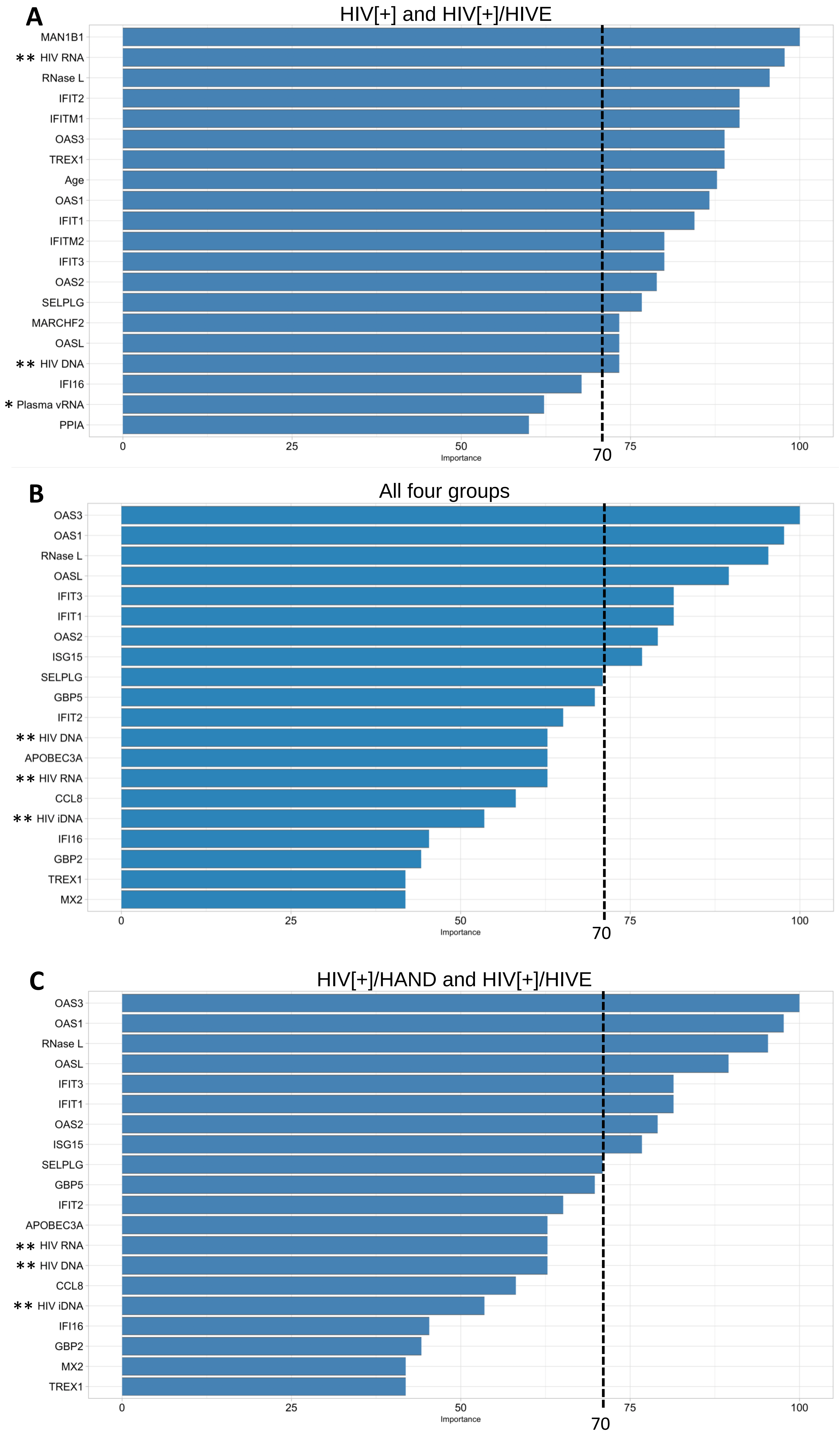
A
HIV[+] and HIV[+]/HIVE
MAN1B1
** HIV RNA
RNase L
IFIT2
IFITM1
OAS3
TREX1
Age
OAS1
IFIT1
IFITM2
IFIT3
OAS2
SELPLG
MARCHF2
OASL
** HIV DNA
IFI16
* Plasma vRNA
PPIA
0
25
50
75
100
Importance
70
B
All four groups
OAS3
OAS1
RNase L
OASL
IFIT3
IFIT1
OAS2
ISG15
SELPLG
GBP5
IFIT2
** HIV DNA
APOBEC3A
** HIV RNA
CCL8
** HIV iDNA
IFI16
GBP2
TREX1
MX2
0
25
50
75
100
Importance
70
C
HIV[+]/HAND and HIV[+]/HIVE
OAS3
OAS1
RNase L
OASL
IFIT3
IFIT1
OAS2
ISG15
SELPLG
GBP5
IFIT2
APOBEC3A
** HIV RNA
** HIV DNA
CCL8
** HIV iDNA
IFI16
GBP2
MX2
TREX1
0
25
50
75
100
Importance
70

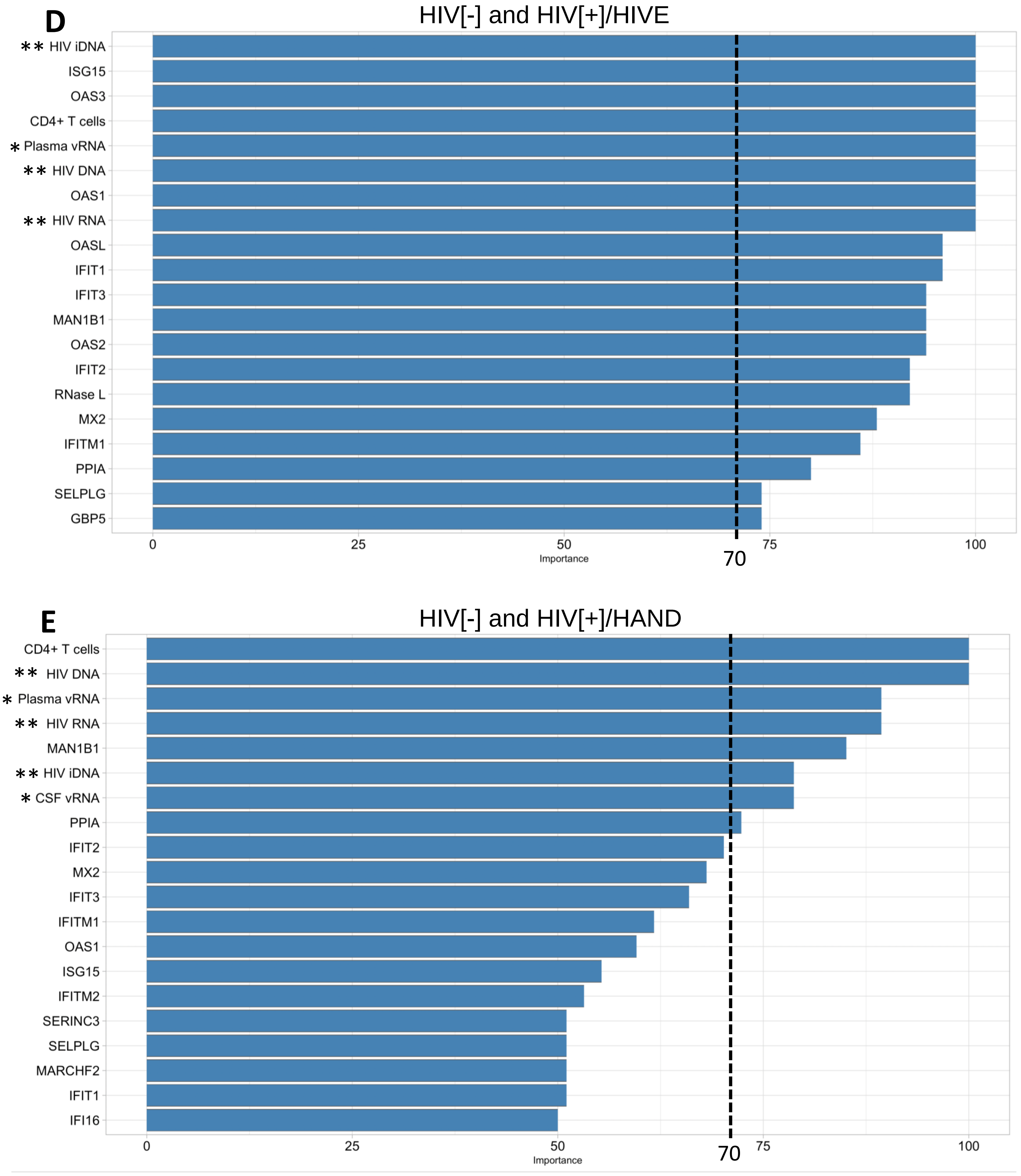

Figure S3. ***Prediction of important variables for group classification using machine learning methods***. Shrinkage discriminant analysis was used to predict variables that distinguished HIV[+] from HIV[+]/HIVE groups (A). Ordinal random forest analysis predicted variable importance among the four groups (B). Shrinkage discriminant analysis was used for prediction of variable importance between HIV[+]/HAND and HIV[+]/HIVE groups (C). Shrinkage discriminant analysis predicted variables that distinguished HIV[-] from HIV[+]/HIVE groups (D). Regularized logistic regression method predicted variables that distinguished HIV[-] from HIV[+]/HAND groups (E). An importance value of 70 was set as a threshold. An importance value between 70-100 indicates heavy reliance of machine learning methods on the specific variable to classify and distinguish the compared groups. (* represents CSF or plasma viral load and ** represents brain viral load (RNA, DNA or iDNA (integrated DNA)).

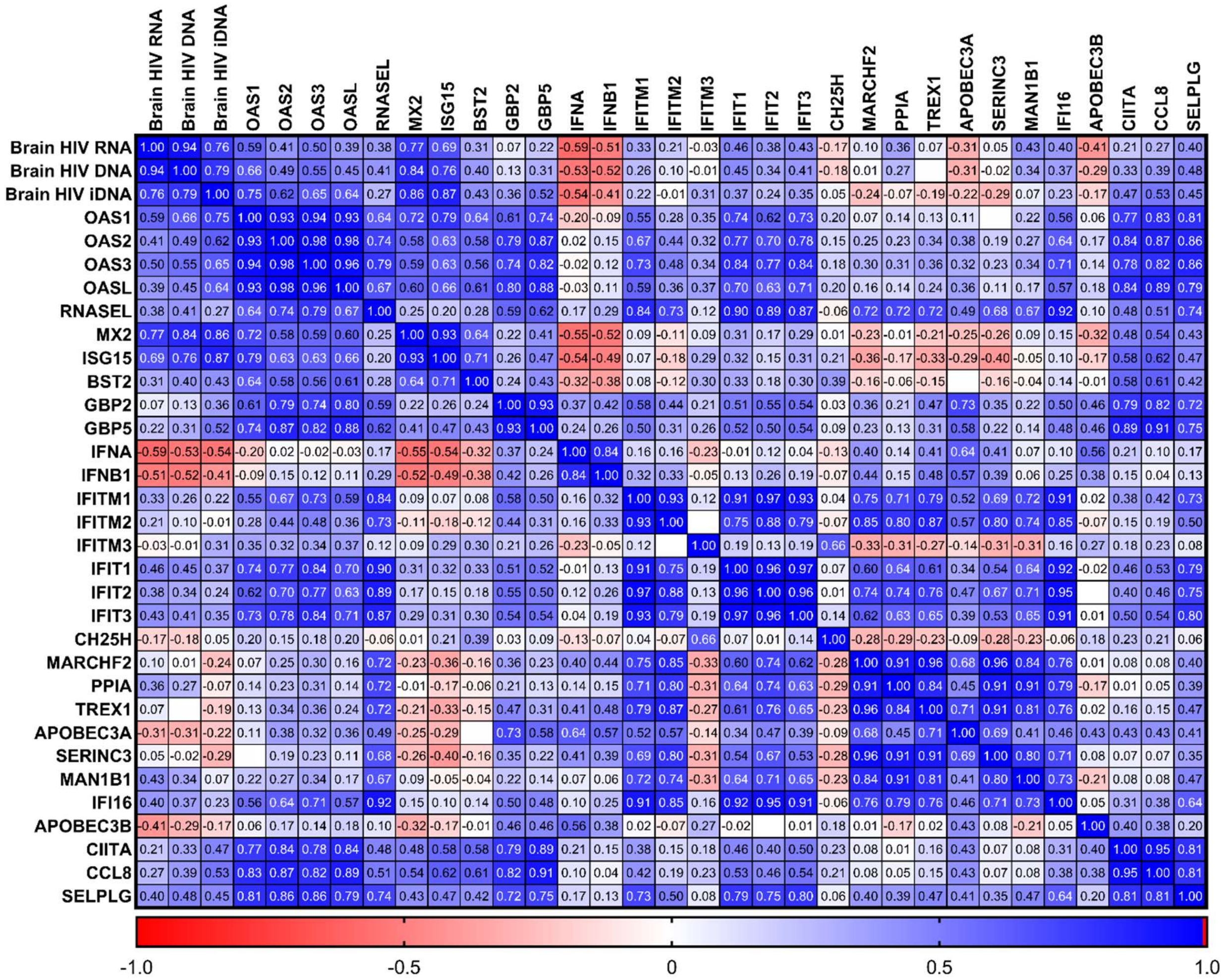

Figure S4. A Spearman correlation matrix of host RFs and brain viral quantities. Correlation coefficients (r) appear in each box Blue-tinted boxes represent positive correlations, while red-tinted boxes represent negative correlations. The boldness of the color represents the strength of the relationship between variables.

Table S1. Oligonucleotide primers for ddPCR and q RT-PCR.

Human primers	Forward 5'-3'	Reverse 5'-3'
HIV-1 pol	TTAAGACAGCAGTACAAATGGCAG	ACTGCCCCTTCACCTTTCCA
Alu	GCCTCCCAAAGTGCTGGGATTACAG	_
HIV-1 gag	_	GTTCCTGCTATGTCACTTCC
OAS1	ATGATGGATCTCAGAAATACCC	TCAGGAACCCACAGATGA
OAS2	ATGGGAAATGGGGAGTCCCA	ACCATCTCGTCGATCAGTGTCT
OAS3	CTTGAGGACTGGATGGATGTTAG	ACTGTTGAGGAGGGTAGAGTAG
OASL	ATGGCACTGATGCAGGAACTGT	TAGCACCTCTTCCTTCCACTCC
RNaseL	CATGGAGAGCAGGGATCATAAC	CTGGACCAGGTCAACATCTTC
GBP2	ATGGCTCCAGAGATCAACTTGC	GGCTGCGTAATTGCAGATAGGA
GBP5	ATGGCTTTAGAGATCCACATGTCA	CCACAACTACAGGTTGCGTAATG
IFIT1	AGAACGGCTGCCTAATTTACAG	GCTCCAGACTATCCTTGACCTG
IFIT2	TGGTGGCAGAAGAGGAAGAT	GTAGGCTGCTCTAAGGAA
IFIT3	GCAGAGACACAGAGGGCAGT	TGGCATTTCAGCTGTGGA
IFITM1	TCAACATCCACAGCGAGACC	TGTCACAGAGCCGAATACCAG
IFITM2	CATCCCGGTAACCCGATCAC	CACGGAGTACGCGAATGCTA
IFITM3	TACTCCGTGAAGTCTAGGGACAG	CAGGTCATGGGCAGAGCTCCTGG
MX2	CCAGAGGCAGCGGAATCGTAACC	GAGCCTTGATCTGCAGTCCGATG
BST2	AGAAGGGCTTTCAGGATGTG	CCTGCTCGGCTTTTCGCTTGAACAT
ISG15	CACAGCCATGGGCTGGGACCTG	CCGGGGCCCAGGCCCTGGCTGGC
IFNα-pan	GTACTGCAGAATCTCTCCTTTCTCCTG	GTGTCTAGATCTGACAACCTCCCAGGCACA
IFNβ1	CTTGGATTCCTACAAAGAAGCAGC	TCCTCCTTCTGGAACTGCTGCA
CIITA	CTACTTCAGGCAGCAGAGGAGA	GCTGTGTCTTCCGAGGAACTTC
CCL8	TATCCAGAGGCTGGAGAGCTAC	TGGAATCCCTGACCCATCTCTC
IFI16	GATGCCTCCATCAACACCAAGC	CTGTTGCGTTCAGCACCATCAC
CH25H	ACTTCTCGCCATCCGCGCAGCAG	CCTTGTGGTGCAGCAGGTGCCAC
MAN1B1	CGTCAACCTGTTTGAGAGCACG	CTTGGATGGTGTTCTGAAGGCAG
SERINC3	CTGATCCTTTGCGTTGTGGCTTC	TTGGACATGGCTGACCAGGTGA
TREX1	CCACTCCTTTCCTTACCACATC	CCACTCCGCCAAACAGAT
PPIA	GGCAAATGCTGGACCCAACACA	TGCTGGTCTTGCCATTCCTGGA
APOBEC3A	GACAATGGCACCTCGGTCAAGA	CCAACTGCAAAGAAGGAACCAGG
MARCHF2	CTAACACCAGCTACTGCGAGCT	GGAAACACACCATGTCGCAGCA
APOBEC3B	CCTCTATGGTCGGAGCTACACT	GAGGAAGCACATTTCTGCGTGG
SELPLG	GAACCTGTCCACGGATTCAGCA	GTCAGTCGAGTTGTCTGTGCCT
**NHP primers**	**Forward 5'-3'**	**Reverse 5'-3'**
SIV pol	GCAGAGGAGGAAATTACCCAGTAC	CAATTTTACCCAGGCATTTAATGT
Alu	TCTGCGTCATCTGGTGCATTCACG	_
SIV gag	_	TGCCAACAGGCTCAGAAAATT
OAS1	ATGATGGATCTCAGAAATACCC	TCAGGAACCCACAGATGA
OAS2	ATGGGAAATGGGGAGTCCCA	ACCATCTCGTCGATCAGTGTCT
OAS3	CTTGAGGACTGGATGGATGTTAG	ACTGTTGAGGAGGGTAGAGTAG
RNaseL	CATGGAGAGCAGGGATCATAAC	CTGGACCAGGTCAACATCTTC
TRIM5a	CTGGAGATGCTGAGGCAGAAGC	GTCCAGGATGTCTCTCAGTTGC
SAMHD1	CTCGCAACTCTTTACACCGTAGA	TTTCCTCCAGCACCTGTAATCTC
IFIT3	GGCAGTTTTTTCCTGTCAGCA	CGTCCTGGCCCATTTCCT
BST2	TCTCCTGCAACAAGAGCTGGCC	TCTCTGCATCCAGGGAAGCCAT
IFNα-pan	TTCCAGCTCTTCAGCACAGA	CTGCATGACACAGGCTTCC
IFNβ1	CTAGCACTGGCTGGAATGAGACT	GGCCTTCAGGTAATGCAGAATC
MX2	CCAGAGGCAGCGGAATCGTAACC	GAGCCTTGATCTGCAGTCCGATG

Table S2. Spearman correlation table for host restriction factors and brain viral load.

	Brain HIV RNA		Brain HIV DNA		Brain HIV iDNA	
	p value	Spearman r	p value	Spearman r	p value	Spearman r
**OAS1**	<0.0005	0.59	<0.00001	0.66	<0.00001	0.75
**OAS2**	<0.05	0.41	<0.005	0.49	<0.0001	0.62
**OAS3**	<0.005	0.50	<0.001	0.55	<0.00005	0.65
**OASL**	<0.05	0.39	<0.01	0.45	<0.0001	0.64
**RNASEL**	<0.05	0.38	<0.05	0.41	ns	0.27
**MX2**	<0.00001	0.77	<0.00001	0.84	<0.00001	0.86
**ISG15**	<0.00001	0.69	<0.00001	0.76	<0.00001	0.87
**BST2**	ns	0.31	<0.05	0.40	<0.05	0.43
**GBP2**	ns	0.07	ns	0.13	<0.05	0.36
**GBP5**	ns	0.22	ns	0.31	<0.005	0.52
**IFNA**	<0.0005	-0.59	<0.005	-0.53	<0.001	-0.54
**IFNB1**	<0.005	-0.51	<0.005	-0.52	<0.05	-0.41
**IFITM1**	ns	0.33	ns	0.26	ns	0.22
**IFITM2**	ns	0.21	ns	0.10	ns	-0.01
**IFITM3**	ns	-0.03	ns	-0.01	ns	0.31
**IFIT1**	<0.01	0.46	<0.01	0.45	<0.05	0.37
**IFIT2**	<0.05	0.38	<0.05	0.34	ns	0.24
**IFIT3**	<0.05	0.43	<0.05	0.41	<0.05	0.35
**CH25H**	ns	-0.17	ns	-0.18	ns	0.05
**MARCHF2**	ns	0.10	ns	0.01	ns	-0.24
**PPIA**	<0.05	0.36	ns	0.27	ns	-0.07
**TREX1**	ns	0.07	ns	0.00	ns	-0.19
**APOBEC3A**	ns	-0.31	ns	-0.31	ns	-0.22
**SERINC3**	ns	0.05	ns	-0.02	ns	-0.29
**MAN1B1**	<0.05	0.43	<0.05	0.34	ns	0.07
**IFI16**	<0.05	0.40	<0.05	0.37	ns	0.23
**APOBEC3B**	<0.05	-0.41	ns	-0.29	ns	-0.17
**CIITA**	ns	0.21	ns	0.33	<0.01	0.47
**CCL8**	ns	0.27	<0.05	0.39	<0.005	0.53
**SELPLG**	<0.05	0.40	<0.005	0.48	<0.01	0.45
